# Cell-based therapies have disease-modifying effects on osteoarthritis in animal models. A systematic review by the ESSKA Orthobiologic Initiative. Part 2: bone marrow-derived cell-based injectable therapies

**DOI:** 10.1007/s00167-023-07320-3

**Published:** 2023-02-24

**Authors:** Angelo Boffa, Carlotta Perucca Orfei, Yosef Sourugeon, Lior Laver, Jérémy Magalon, Mikel Sánchez, Thomas Tischer, Laura de Girolamo, Giuseppe Filardo

**Affiliations:** 1 Applied and Translational Research Center, IRCCS Istituto Ortopedico Rizzoli, Bologna, Italy; 2 Clinica Ortopedica e Traumatologica 2, IRCCS Istituto Ortopedico Rizzoli, Bologna, Italy; 3 Laboratorio di Biotecnologie Applicate all’Ortopedia, IRCCS Ospedale Galeazzi Sant’Ambrogio, Via Cristina Belgioioso 173, 20157 Milan, Italy; 4 Rambam Health Care Campus, Haifa, Israel; 5 Department of Orthopaedics, Hillel Yaffe Medical Center (HYMC), Hadera, Israel; 6 Arthrosport Clinic, Tel‑Aviv, Israel; 7 Rappaport Faculty of Medicine, Technion University Hospital (Israel Institute of Technology), Haifa, Israel; 8 Cell Therapy Laboratory, Hôpital De La Conception, AP-HM, Marseille, France; 9 INSERM, NRA, C2VN, Aix Marseille Univ, Marseille, France; 10 SAS Remedex, Marseille, France; 11 Arthroscopic Surgery Unit, Hospital Vithas Vitoria, Vitoria‑Gasteiz, Spain; 12 Advanced Biological Therapy Unit, Hospital Vithas Vitoria, Vitoria‑Gasteiz, Spain; 13 Department of Orthopaedic Surgery, University of Rostock, Rostock, Germany; 14 Department of Orthopaedic and Trauma Surgery, Malteser Waldkrankenhaus St. Marien, Erlangen, Germany; 15 Service of Orthopaedics and Traumatology, Department of Surgery, EOC, Lugano, Switzerland; 16 Faculty of Biomedical Sciences, Università Della Svizzera Italiana, Lugano, Switzerland

**Keywords:** Mesenchymal stromal cells (MSCs), Stem cells, Bone marrow, Intra-articular, Injection, Disease-modifying, Osteoarthritis, Cartilage

## Abstract

**Purpose:**

Aim of this systematic review was to determine if bone marrow-derived cell-based injectable therapies induce disease-modifying effects in joints affected by osteoarthritis (OA) in animal models.

**Methods:**

A systematic review was performed on three electronic databases (PubMed, Web of Science, Embase) according to PRISMA guidelines. A synthesis of the results was performed investigating disease-modifying effects in preclinical animal studies comparing injectable bone marrow-derived products with OA controls or other products, different formulations or injection intervals, and the combination with other products. The risk of bias was assessed according to the SYRCLE’s tool.

**Results:**

Fifty-three studies were included (1819 animals) with an increasing publication trend over time. Expanded cells were used in 48 studies, point-of-care products in 3 studies, and both approaches were investigated in 2 studies. Among the 47 studies presenting results on the disease-modifying effects, 40 studies (85%) reported better results with bone marrow-derived products compared to OA controls, with positive findings evident in 14 out of 20 studies (70%) in macroscopic assessment, in 30 out of 41 studies (73%) in histological assessment, and in 10 out of 13 studies (77%) in immunohistochemical evaluations. Clinical evaluations showed positive results in 7 studies out of 9 (78%), positive imaging results in 11 studies out of 17 (65%), and positive biomarker results in 5 studies out of 10 (50%). While 36 out of 46 studies (78%) reported positive results at the cartilage level, only 3 out of 10 studies (30%) could detect positive changes at the synovial level. The risk of bias was low in 42% of items, unclear in 50%, and high in 8%.

**Conclusion:**

This systematic review of preclinical studies demonstrated that intra-articular injections of bone marrow-derived products can induce disease-modifying effects in the treatment of OA, slowing down the progression of cartilage damage with benefits at macroscopic, histological, and immunohistochemical levels. Positive results have been also observed in terms of clinical and imaging findings, as well as in the modulation of inflammatory and cartilage biomarkers, while poor effects have been described on the synovial membrane. These findings are important to understand the potential of bone marrow-derived products and to guide further research to optimise their use in the clinical practice.

**Level of evidence:**

II.

**Supplementary Information:**

The online version contains supplementary material available at 10.1007/s00167-023-07320-3.

## Introduction

Osteoarthritis (OA) is the most common form of joint degenerative disease and one of the major causes of pain and disability in older adults, with a heavy burden on healthcare systems [1–3]. Current management strategies to address OA joints [4] can provide only a partial symptom relief with a short-term effect, rather than disease-modifying changes [5]. Therefore, there is the need to identify novel strategies to reduce the progression of this common and debilitating condition [6, 7]. In this scenario, the use of orthobiologics is gaining increasing interest due to their mechanisms of action [5], and recently, in the previous ESSKA Orthobiologic Initiative (ORBIT) analysis of platelet-rich plasma (PRP) literature, PRP injections showed disease-modifying effects in OA animal models [8]. The use of mesenchymal stromal cells (MSCs) has also been proposed as a valid option for OA treatment thanks to their potential disease-modifying effects, as underlined for adipose-derived products in the cell-focused ORBIT literature analysis Part 1 [9].

Bone marrow was the first source of MSCs (BMSCs) and, due to the ease of collection, it is nowadays commonly used to isolate MSCs [10]. This type of MSCs has been either applied after in vitro culture expansion or used as bone marrow aspirate concentrate (BMAC) in one-step treatments [10]. In vitro studies encouraged the use of these products for the repair of damaged cartilage and OA treatment, given the self-renewal characteristics, the multidifferentiation ability, and the paracrine effects of BMSCs [11, 12]. Intra-articular use of bone marrow-derived products has been the subject of many clinical trials for the last decade [13–20]. Nevertheless, their real therapeutic potential as intra-articular OA treatment remains controversial and, due to ethical and practical concerns and national regulatory frameworks, the evidence of possible disease-modifying effects of these products in the clinical setting is even more limited. To this aim, animal models can help understanding the mechanism of action and potential disease-modifying effects of this biological approach, guiding its translation in the clinical practice.

The purpose of this systematic review, part 2 of a series of publications by the ESSKA ORBIT on the use of cell-based products for the treatment of OA, was to investigate in the animal models the presence of disease-modifying effects driven by bone marrow-derived products for the intra-articular injective treatment of OA.

## Materials and methods

### Search strategy and article selection

A systematic review of the literature was performed on three electronic databases (PubMed, Web of Science, and Embase) according to the Preferred Reporting Items for Systematic Reviews and Meta-Analyses (PRISMA) guidelines. The methodology of this systematic review, divided into three articles (according to different MSCs sources), was already reported in the previous publication [9]. Two authors (CP and YS) conducted screening process and analysis, and a third author (AB) was involved to resolve any discrepancies. Preclinical studies focusing on the intra-articular use of MSCs to address joints affected by OA were included based on the following inclusion criteria: Animal studies, articles written in English, purely injective treatments for cartilage degeneration and OA. Exclusion criteria were: In vitro or clinical studies, congress abstracts, reviews, articles written in other languages than English, studies on joint diseases different from OA, studies analysing associated surgery, studies on the use of MSC secretome/extracellular vesicles, and studies reporting the use of MSCs without a control group or the combined use of MSCs with another product without analysing the specific contribution of MSCs application.

**Fig. 1:**
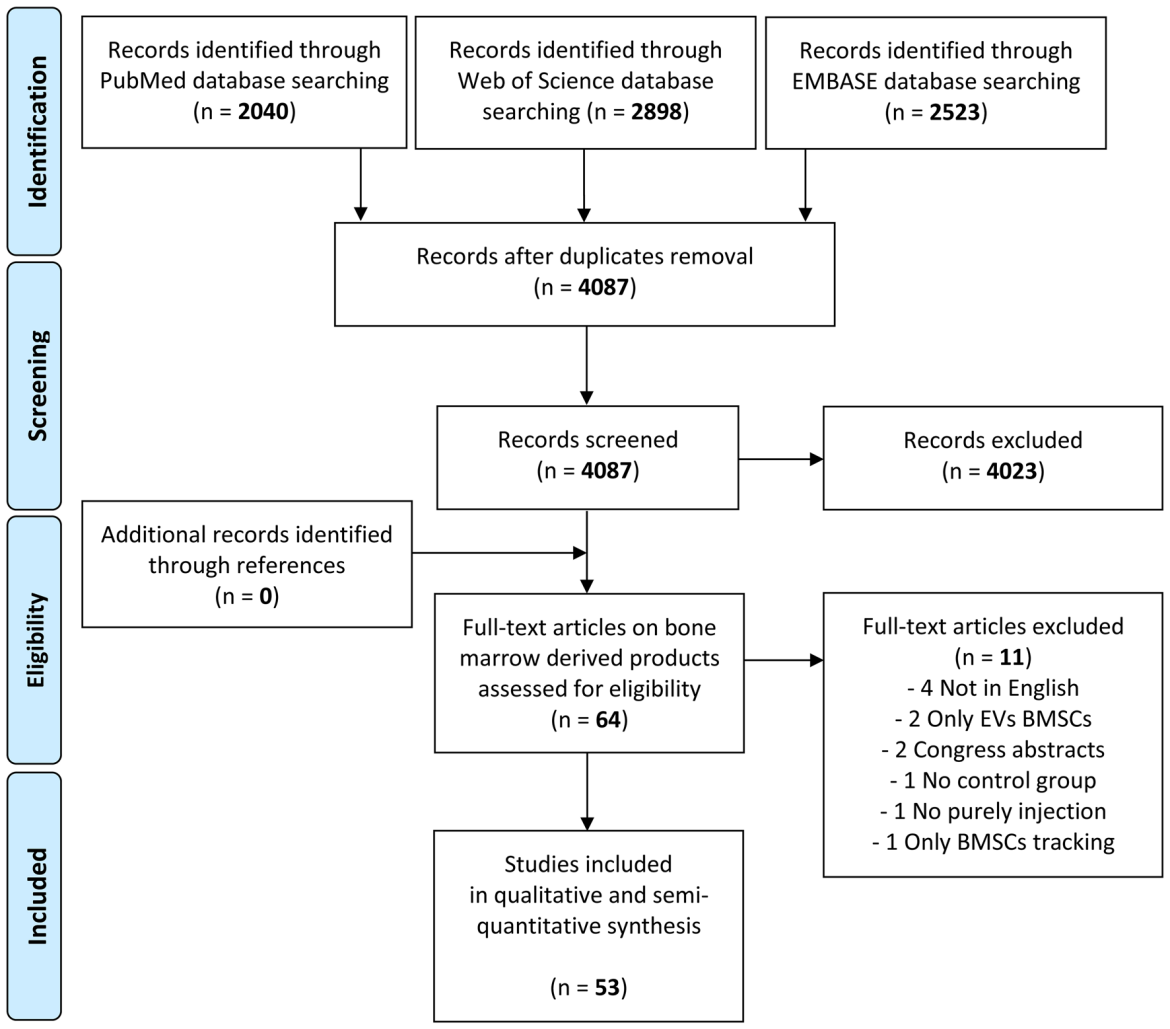
PRISMA flowchart of the study selection process. *BMSCs* bone marrow mesenchymal stromal cells; *EVs* extracellular vesicles

While the first article of this ORBIT series analysed the disease-modifying effects of adipose tissue-derived products [9], the current manuscript focuses on bone marrow-derived products. The flowchart reported in Fig. 1 describes graphically the systematic review process.

### Data extraction and quality assessment

Relevant data were extracted from article texts, tables, and figures and then collected in a database to be analysed for the purpose of the present study. In detail, the following data were collected: authors, journal, year of publication, number and type of evaluated animals, joint involved, OA model, type of treatment, follow-up, results, and bone marrow-derived products characteristics including source, origin, MSC count, additional procedures, processing modality (expanded versus “point-of-care”), and injective protocol. A synthesis of the obtained results was performed analysing the clinical and imaging findings as well as the disease-modifying effects on the OA process of the different preparations. The effects provided by the bone marrow-derived product injections were considered positive in a disease-modifying perspective when the study reported better objective results (with a statistical significance) for cell therapies compared to OA controls in at least one of the following outcomes: macroscopic, histological, and/or immunohistochemical findings. In particular, the analysis was based on the comparison of the experimental groups versus the respective controls (vehicle injection or no treatment). Moreover, other results were analysed when available regarding the benefits provided by different doses or injection schedules, the effects versus other injectable treatments, and finally the effects derived from the combination of bone marrow-derived products with other products exploring potential synergistic effects. The risk of bias assessment of the included studies was performed using the Systematic Review Centre for Laboratory animal Experimentation (SYRCLE)’s tool [21].

## Results

### Study selection and analysis

**Fig. 2:**
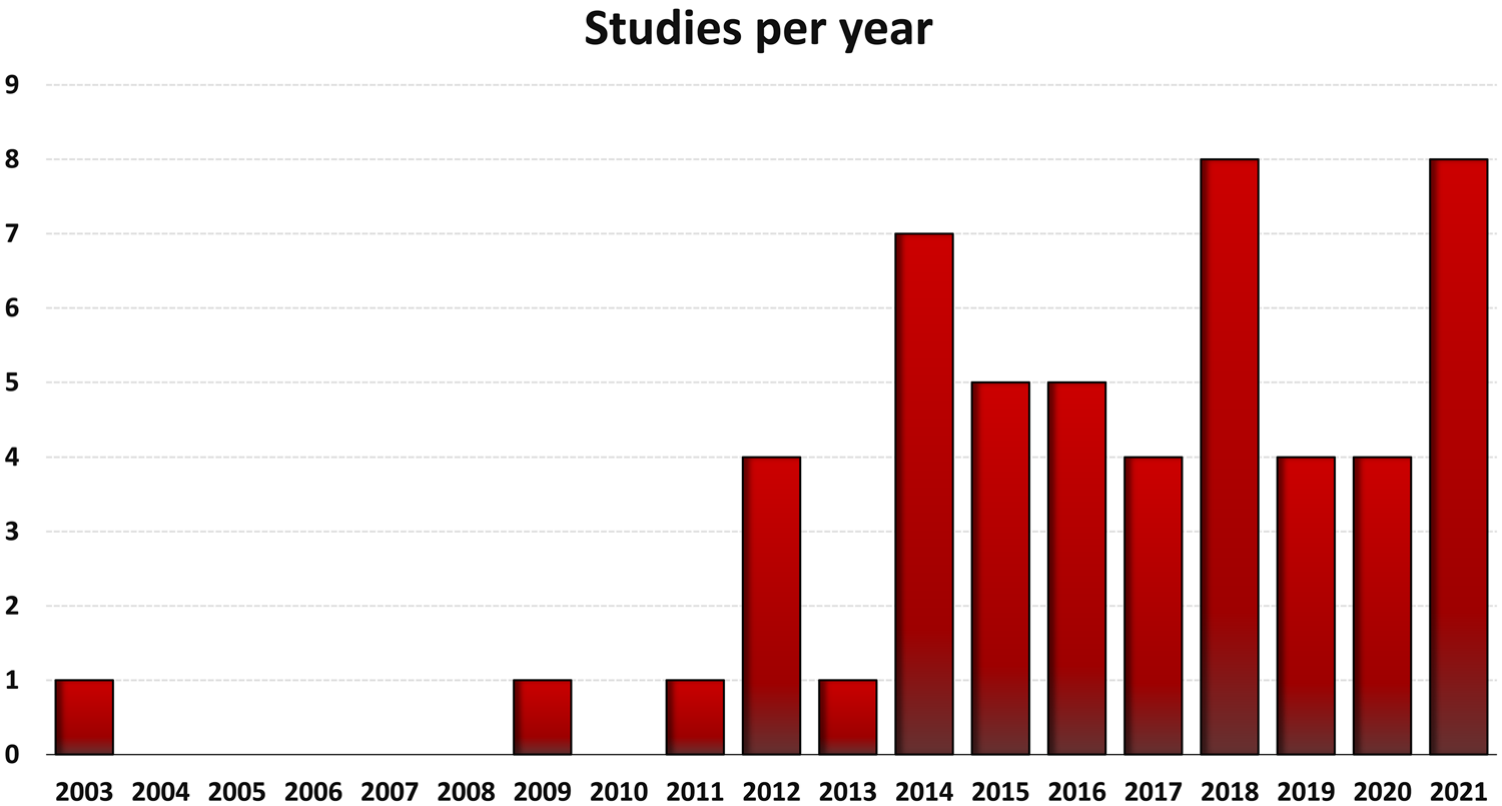
Animal studies on intra-articular injections of bone marrow-derived products to address OA over the years

From the 4087 items obtained from the initial search, a total of 53 studies focusing on bone marrow-derived products were included in the qualitative data synthesis (Fig. 1). From the first report published in 2003, the publication trend increased over the years, with over 80% of the included studies published from 2014 (Fig. 2).

Thirty-seven studies analysed the effects on small animal OA models (rodents: 28; rabbits: 9), while 16 studies focused on large animals (sheep: 7; horses: 4; goats: 2; monkeys: 2; pig: 1). A total of 1,819 animals were assessed: 1,317 rodents, 235 rabbits, 101 sheep, 77 horses, 48 goats, 35 monkeys, and 6 pigs. The treated joints were knees in most studies (48 articles), carpal joints in 3 studies, fetlock joints in 1 study, and temporo-mandibular joints (TMJ) in 1 study. The OA model was surgically induced in 35 studies (mostly through meniscectomy and/or ligament transection), chemically induced in 12 studies (through the injection of chondrotoxic or pro-inflammatory products), naturally occurring in 2 studies (veterinary studies), induced by joint immobilisation in 2 studies, induced by a closed tibial plateau fracture in 1 study, and induced by a unilateral anterior crossbite prosthesis [22] in the only study on the TMJ.

Expanded BMSCs were analysed in 48 studies, “point-of-care” bone marrow-derived products were used in 3 studies, while other 2 studies evaluated both expanded BMSCs and “point-of-care” products. All the studies on expanded BMSCs described the injected MSCs dose, which ranged from 2.0 × 10^3^ to 1.0 × 10^7^ for small animals and from 0.7 × 10^6^ to 4.5 × 10^8^ for large animals. Only two out of the 5 studies on “point-of-care” bone marrow products reported the injected MSC dose, which was 1.0 × 10^6^ in both cases. Allogeneic bone marrow products were used in 26 studies, 15 studies used autologous products, 10 studies used xenogeneic products, 9 of them using human-derived BMSCs. Two studies did not specify the MSC origin. The bone marrow harvest site was described in 38 studies: iliac crest in 14 studies, both femur and tibia in 10 studies, femur in 5 studies, sternum in 4 studies, humerus in 2 studies, tibia in 2 studies, and both femur and humerus in 1 study. Additional procedures to expanded BMSCs were described in 20 studies, including preconditioning with vitamin E or kartogenin, induction of overexpressed transcription factors, expansion under hypoxic condition, chondrogenic differentiation, or encapsulation in microsphere. The amount of the injected volume ranged from 4.0 μL  to 2.0 mL in small animals and from 0.1 mL to 5.0 mL in large animals, while the injective protocol consisted of a single injection in 41 studies or multiple injections (from 2 to 7) in the other 12 studies. The follow-up period of the included studies ranged 2 weeks–12 months following OA induction. Further details on the characteristics of the included studies are reported in Supplementary Table 1.

### Disease-modifying effects on OA joints

**Fig. 3:**
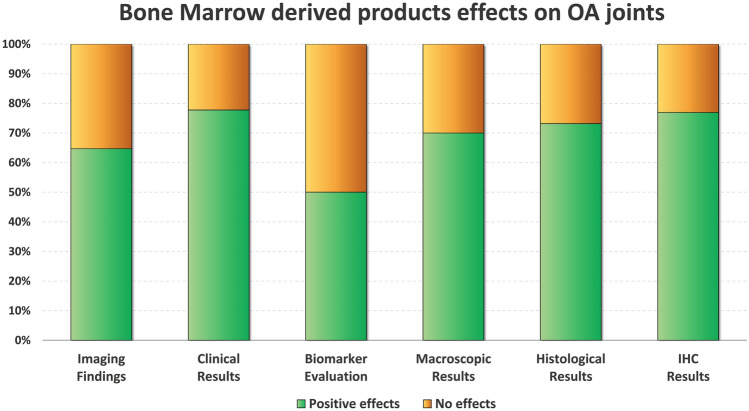
Disease-modifying effects on OA joints induced by bone marrow-derived products. The bar chart shows the percentage of studies that met the specific effects. Positive effects (green) vs no effects (orange) in imaging findings (*n* = 16), clinical results (*n* = 9), biomarker evaluation (*n* = 10), macroscopic results (*n* = 20), histological results (*n* = 21) and immunohistochemical results (*n* = 13). *IHC* immunohistochemistry; *OA* osteoarthritis

Forty-seven studies out of 53 investigated the disease-modifying effects of bone marrow-derived products injections with respect to OA controls (vehicle injections or untreated joints). Overall, 40 studies (85.1%) documented better results compared to OA controls in at least one of the following outcomes: macroscopic, histological, and/or immunohistochemical findings, while the remaining 7 studies (14.9%) reported no improvement from the injective treatment. In particular, 14/20 studies (70.0%) with macroscopic evaluations (gross morphological scores) documented overall better results, 30/41 studies (73.2%) with histological evaluations reported overall better results, and 10/13 studies (76.9%) with immunohistochemical analyses revealed overall better results after the injections with bone marrow-derived products. The comparison between small and large animal OA models showed similar overall positive disease-modifying effects (84.8% and 85.7%, respectively). A more detailed analysis is reported in the following paragraphs and in Fig. 3.

### Disease-modifying effects at cartilage level

A total of 46 studies investigated the disease-modifying effects at the cartilage level. Among these, 36 studies (78.3%) reported positive results. In detail, less articular degeneration was macroscopically observed, with joints showing better cartilage volume and thickness, relatively smooth articular surface, and less osteophytes formation versus OA controls [23–32]. Histological analysis documented a higher number of chondrocytes with lower apoptosis ratio and better cell organisation and density, with abundant extracellular matrix, better proteoglycan content, and higher glycosaminoglycan content compared to OA controls [11, 22, 26–28, 32–36]. Immunohistochemical evaluations revealed an increased expression of aggrecan and collagen type-II alpha and a decreased expression of collagen type-I alpha [11, 33, 35, 37]. Finally, bone marrow-derived products induced in chondrocytes a higher mRNA/gene expression of type-II collagen, aggrecan, B-cell lymphoma 2 (BCL-2), cyclin D1, proliferating cell nuclear antigen (PCNA), transforming growth factor beta (TGF-β), and tissue inhibitor of metalloproteinases 1 (TIMP-1), and a lower mRNA/gene expression of type-I collagen, Adamts-4, BCL-2-like protein 4 (Bax), Caspase-3, cyclooxygenase 2 (COX-2), interleukin-1 beta (IL-1β), matrix metalloproteinase 1 (MMP-1), MMP-4, MMP-13, nuclear factor-kappa B (NF-Κb) p65, protein P21, and vascular endothelial growth factor VEGF [22, 24, 33, 38–42].

### Disease-modifying effects at synovial membrane level

Only 3 of the 10 studies investigating the disease-modifying effects on the synovial membrane documented positive results, while the others 7 showed no differences compared to OA controls. Benefits in the synovial membrane were observed in terms of vascularity, subintimal fibrosis and oedema, and cellular infiltration, indicating a decreased inflammation and a reduction of hypertrophic, fibrotic, and angiogenesis processes. Moreover, BMSCs injections decreased MMP-1 and MMP-13 expression, downregulated IL-1β, TIMP-2, and tumour necrosis factor alpha (TNF-α), and increased ratios of homeostatic macrophages [24, 35, 43].

### Effects on OA biomarkers profile

Ten studies investigated indirect disease-modifying effects through the measurement of serum or synovial fluid biomarkers related to cartilage metabolism or inflammation. Half of these studies reported positive results compared to OA controls, documenting an increase of the serum levels of IL-10 and a decrease of the synovial fluid levels of IL-1β, IL-6, MMP-1, MMP-13, nitric oxide (NO), prostaglandin E2 (PGE2), TGF-β, and TNF-α [28, 32, 39, 44, 45].

### Clinical effects

Nine studies quantitatively evaluated the effects on clinical parameters compared to OA controls. Of these, 7 studies (77.8%) documented better clinical outcomes reporting reduction of joint swelling, increase of joint flexibility, reduction of tactile allodynia, increase of the weight-bearing on the injured leg, and a lower paw withdrawal threshold [24, 40, 41, 46–49].

### Imaging analysis

Seventeen studies performed imaging analyses to detect the effects on the injected joints. Among these, 11 (64.7%) reported significantly improved effects compared to OA controls. In detail, a micro-computed tomography (micro-CT) analysis was performed in 9 studies, with 7 reporting positive results in preserving the microstructural changes in subchondral bone induced by the OA process. In particular, an increase of bone trabeculae, trabecular bone volume fraction, and bone mineral density was found [11, 22, 25, 44, 50–52]. Radiographic evaluation was conducted in 6 studies, with 4 finding no differences and only 2 able to demonstrate benefits from cell therapy in terms of less severe radiological signs of OA (including osteophyte formation, subchondral bone sclerosis, and articular surface irregularity) compared to controls [53, 54]. Magnetic resonance imaging (MRI) assessment was performed in 5 studies, with 3 finding no differences and only 2 reporting positive results, with less severe MRI signs of OA and less evidence of inflammation in terms of hyperintense fluid accumulation compared to OA controls [43, 53]. Finally, one study performed an ultrasonographic evaluation documenting the decrease of joint effusion and capsulitis [24].

### Comparison of formulations and protocols

Twenty studies compared the disease-modifying effects of bone marrow-derived products differing in dose, preparation, injective protocol, or additional procedures/pre-treatments. In detail, 8 studies evaluated the benefits offered by different additional procedures, reporting better results compared to BMSCs alone in case of preconditioning with Kartogenin or Vitamin E, supplementation with curcumin, transfection with transforming growth factor receptor I (ALK5) plasmid or adenoviral vectors expressing vIL-10 (AdIL-10), and pre-treatment with the autophagy inhibitor 3-Methyladenine or stimulation with low-intensity pulsed ultrasound [11, 33, 37, 39, 49, 55, 56]. No overall advantages were observed in case of priming with TNF-α and interferon-γ (IFN-γ) [24].

Four studies evaluated the effects of BMSC encapsulation in microspheres. Of these, one study [52] documented better chondroprotective therapeutic effect after encapsulated BMSC injections compared to the BMSCs alone, while two other studies [48] did not show an overall superiority of encapsulated BMSCs. Other authors [57] compared the effects of BMSCs encapsulated in microspheres releasing or not TGFβ3, documenting similar positive effects between the two treatments.

Three studies compared culture-expanded BMSCs with non-expanded bone marrow-derived products. The first [35] did not report any significant difference between expanded BMSCs and BMAC in terms of disease-modifying effects on cartilage, synovial membrane, and meniscus, similar to another study [58] which reported no differences between expanded BMSCs and cells obtained using a Ficoll gradient in terms of cartilage and subchondral bone damage protection, and synovial inflammation improvement. On the other hand, other authors [28] demonstrated higher cartilage regeneration and lower proteoglycan loss in animals treated with expanded BMSCs compared to non-expanded BMCSs obtained via density gradient centrifugation.

Two studies analysed the role of chondrogenic differentiation of BMSCs before injection, with one [26] reporting better histological findings with chondrogenic differentiated BMSCs compared to undifferentiated ones, and the other [23] observing significant benefits after chondrogenic differentiation in terms of reduction of meniscus damage.

One study evaluated two different BMSC injective protocols comparing single injection versus three injections in a rabbit OA model. The authors found that multiple injections induced better results in terms of macroscopic, histological, and immunohistochemical findings [27].

One study compared the effects of different BMSC doses on OA joints, revealing that the therapeutic effect depended on the number of cells applied to animals, with the best effects observed with high dose formulations [59].

Finally, one study compared the effects of early versus late passage BMSCs, reporting better results in terms of pain improvement in animals treated with late passage BMSCs [46].

### Comparison with other injectable products

Sixteen studies compared the disease-modifying effect of bone marrow-derived products with other injectable products. In detail, 5 studies evaluated animals treated with BMSCs versus adipose-derived stem/stromal cells (ASCs), with 4 studies reporting comparable results in terms of clinical improvement, macroscopic and histological findings, while one study documented better mechanical properties in cartilage compression tests after BMSCs injection [29–31, 45, 60].

Five studies compared bone marrow-derived products versus hyaluronic acid (HA) injections reporting controversial results. One study [61] documented better histological results at cartilage level after BMSCs injections compared to viscosupplementation, while three studies did not find any difference in terms of macroscopic, histological, and immunohistochemical findings [35, 62, 63]. One of these studies [35] also compared BMAC versus HA, reporting similar histological and immunohistochemical results, although BMAC provided a higher meniscal type-II collage expression. Finally, Shu et al. [41] documented better results for HA versus BMSCs injection in terms of tactile allodynia reduction.

Two studies compared the effects of BMSCs versus exosomes isolated from conditioned medium of BMSCs, documenting equal potential in the protection from joint damage [25, 50]. Other two studies compared the injections of BMSCs versus umbilical cord blood-derived MSCs or embryonic stem cells (hESCs) spheroids, without observing significant differences between MSC sources in terms of histopathological results, imaging findings, and synovial fluid parameters [47, 53]. In another study the authors compared BMAC versus PRP injections, reporting more favourable macroscopic and histological findings in joints treated with BMAC, with greater cartilage protection and less extracellular matrix loss [32]. Finally, one study comparing BMSCs versus corticosteroids injections, reported higher inhibition of weight-bearing asymmetry in animals treated with steroids, while no significant differences between the two injectable treatments were found in terms of chondropathy score, synovitis score, and levels of pain-related cytokines [46].

### Effects of injectable combined treatments

Twelve studies evaluated the synergistic effects of bone marrow-derived products when used combined with other injectable options. No studies reported a higher number of complications with the combined use versus the single use of the injectable products. Eight studies evaluated the disease-modifying effects of the combined use of bone marrow-derived products with HA compared to viscosupplementation alone, reporting controversial results. Four studies showed better macroscopic, histological, and/or immunohistochemical findings with the combined use of BMSCs and HA compared to HA alone [62, 64–66]. One study indicated less clear results comparing the synergistic effects of BMSCs or BMAC with HA injections, reporting that BMAC-HA treatment resulted as the best strategy to support joint repair, although the only significant difference with respect to HA alone was observed at histological evaluation of the meniscal tissue [35]. Two studies did not report any benefits from the combined use of BMSCs and HA compared with HA alone [63, 67], while one study reported worse results for this combination in terms of synovitis grade [41].

Two studies investigated the disease-modifying effects of the combined use of bone marrow-derived products with PRP, reporting no statistically significant differences both for BMSCs and BMAC versus PRP treatment alone [67, 68].

One study reported that the combination of BMSCs and cultured articular cartilage chondrocytes (ACCs) attenuated the severity of OA cartilage degeneration better than ACC treatment alone [61].

Finally, one study demonstrated that the combined use of BMSCs and Lugua polypeptide injections had lower synovial fluid level of inflammatory biomarkers compared to Lugua polypeptide injection alone [39].

### Risk of bias assessment

**Fig. 4:**
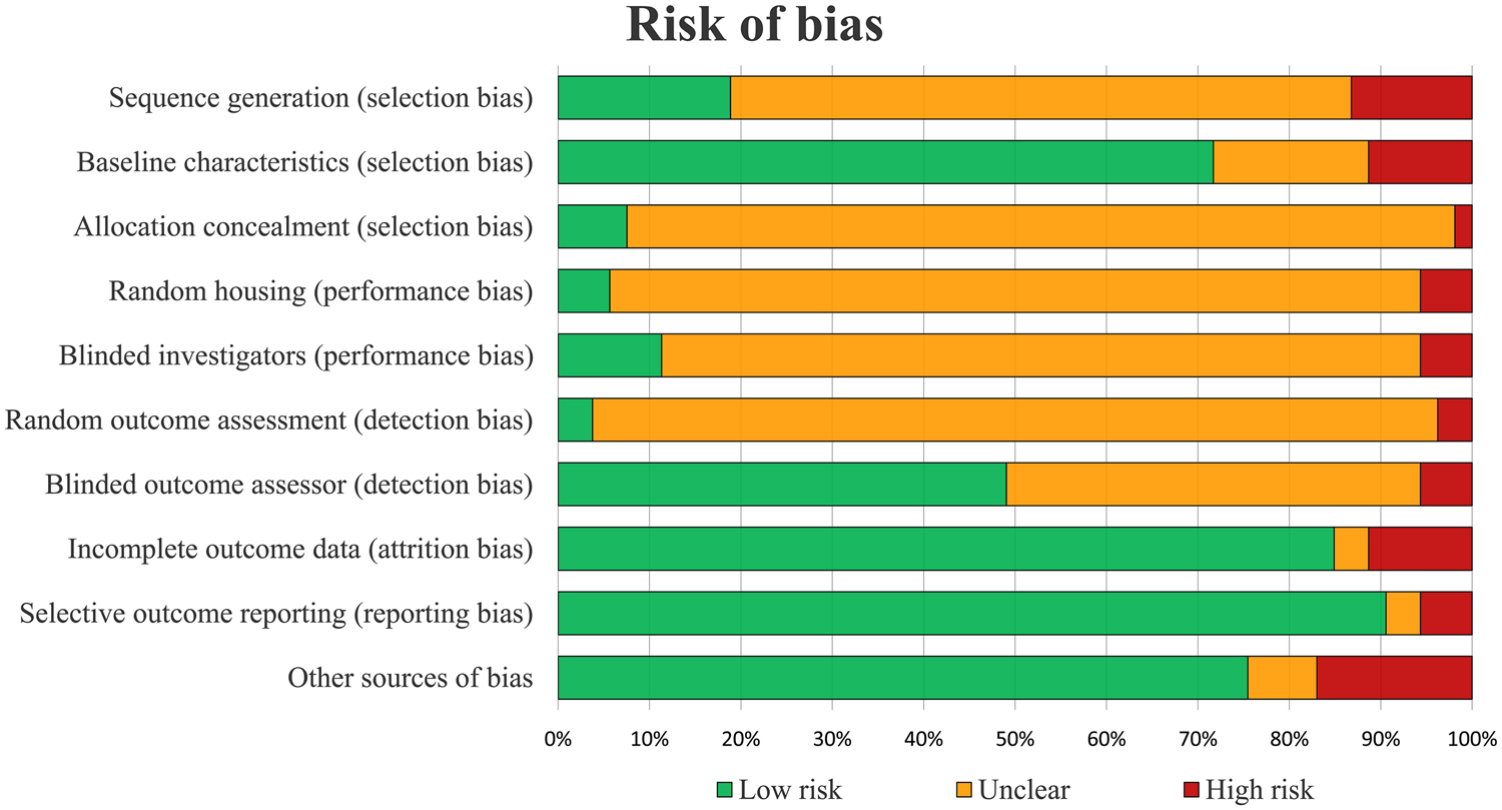
SYRCLE's risk of bias tool assessment of the included studies. The bar chart shows the percentage of all studies that met each quality item, scored as “Low risk”, “High risk”, or “Unclear”

Risk of bias assessment of all included studies is illustrated in Fig. 4. There was a 82% agreement between the two authors involved in the evaluation of the risk of bias. Half of items (50%) were rated as unclear, while low and high risk of bias were observed in 42% and 8%, respectively. The evaluation of risk of bias over time did not show a significant trend towards improving the quality of the included studies, with low-risk items reported in 44% vs 38% in the most recent half of the papers vs the older ones.

## Discussion

The main finding of this systematic review of preclinical studies is that the injections of bone marrow-derived products induce disease-modifying effects in OA joints in at least one of the related outcomes in 85% of the studies, slowing down the progression of cartilage damage with benefits observed at macroscopic, histological, and immunohistochemical levels, and with positive effects in terms of clinical and imaging findings. Less positive benefits were reported on the synovial membrane and half of the studies detected positive changes in serum and synovial fluid levels of inflammatory and cartilage biomarkers.

Bone marrow is one of the most common sources of MSCs, thanks to its relative accessibility, availability, and the potential for point-of-care use [5]. It contains a heterogeneous mixture of mature cells as well as a much smaller population of MSCs and progenitor cells that support the preservation and homeostasis of the tissue over time [5, 69, 70]. These cells have been widely used for cartilage tissue pathologies due to their self-renewal and differentiating properties into chondrogenic lineages [71]. Recently, increasing evidence indicates that the biological and therapeutic effects of these MSCs are mostly attributed to their paracrine mechanisms, rather than direct differentiation into the target tissue, mainly via secreting several molecules, including growth factors, chemokines, cytokines, and extracellular vesicles [72, 73] able to promote tissue healing and modulate the inflammatory process [12, 74, 75].

These BMSCs properties have shown the potential to positively affect the cartilage surface, as shown by in vitro studies demonstrating the promotion of the repair of damaged cartilage by differentiating into chondrocytes, as well as by inducing proliferation and maturation of the remaining healthy chondrocytes or by inducing differentiation of chondroprogenitors [76]. These positive results have been also confirmed in the current systematic review of preclinical animal studies, where 78% of the studies reported benefits at the cartilage level in animal models treated with bone marrow-derived products compared to controls. These benefits have been observed at macroscopic, histological, and immunohistochemical levels, with positive disease-modifying effects in 70%, 73%, and 77% of the studies, respectively. Interestingly, more sensitive analyses allowed to detect positive effects that could not be observed with less accurate modalities. In fact, imaging evaluations like MRI and radiographs documented disease-modifying effects after cell therapy only in 4 out of 9 studies, often reporting no differences with OA controls. This confirms the limits of the imaging evaluations in detecting changes at cartilage and subchondral bone levels, as already documented in the clinical practice, where there is poor correlation between clinical outcome and imaging findings [77]. A similar trend was previously observed for adipose-derived products, with immunohistochemical analyses resulting the method able to underline more disease-modifying effects, followed by histological, macroscopic, with imaging evaluations being less useful [9].

The comparison with the results obtained by the ESKKA ORBIT systematic review on adipose-derived products showed a similar clinical improvement for both cell therapies, with 78% of the studies reporting clinical benefits compared to OA controls in both cases. However, different findings could be observed in terms of disease-modifying properties. Adipose-derived products showed a higher percentage of overall disease-modifying effects compared to bone marrow-derived products (94% vs 85%). More specific evaluations further strengthen the observed difference, at macroscopic (85% vs 70%), histological (93% vs 73%), and immunohistochemical (96% vs 77%) levels. This was confirmed at cartilage (96% vs 78%), biomarker (86% vs 50%), and synovial levels (60% vs 30%).

The comparison at the biomarker and synovial levels is particularly interesting, as it shows on one side a marked difference between the cell sources, and on the other hand an overall poor potential of bone marrow-derived products in modulating the OA inflammatory environment. To this regard, previous in vitro studies suggested a different behaviour of human MSCs derived from bone marrow and adipose tissue in an OA microenvironment, with a worse response of BMSCs versus ASCs to the inflammatory conditions [78–80] The possible lower capacity of BMSCs to respond to the “inflammatory attack” [79] may have important clinical implications, considering the challenging environment of the OA joints in the clinical practice. Still, these findings should be confirmed by more targeted studies directly comparing the two approaches in in vivo conditions. In fact, only 5 studies (on expanded cell products) directly compared the two autologous sources of MSCs in animal models, with 4 of them reporting comparable results in terms of clinical improvement, macroscopic, and histological findings, while one study documented even better mechanical properties in cartilage compression tests after BMSCs injection compared to ASCs. These results are in line with the only available clinical study that directly compared a bone marrow-derived product (BMAC) versus an adipose-derived product in humans (micro-fragmented adipose tissue), without finding significant difference in clinical improvement after the injective procedure [18]. These results seem in contrast with the overall better findings in the preclinical literature of adipose-derived products, which were not able to translate in clear evidence of superior disease-modifying effects and clinical benefits. This underlines the need for better comparative evaluations in more suitable preclinical models as well as in rigorous clinical trials. Future studies should investigate pros and cons of the two tissue sources and directly compare their results in the clinical setting to identify the best cell therapy for OA management.

Despite the recent growing interest towards other MSCs sources showing more promising features in terms of results and availability, this systematic review showed that the research on bone marrow-derived products continues to be active, with an increasing trend in publications of preclinical animal studies on this topic observed over the years. However, these preclinical study findings do not directly translate into treatments for the clinical practice, since preclinical and clinical studies on bone marrow-derived products did not show the same focus. In fact, while preclinical studies are focused mostly on cultured expanded BMSCs (48 out of 53 studies) and only 5 studies evaluated point-of-care injections, BMAC is more commonly used and investigated in the clinical setting. A recent systematic review of the literature found 18 clinical studies evaluating the effects of BMAC for the treatment of OA joints, with promising results in terms of safety and effectiveness [15]. However, the current knowledge is still preliminary, considering the high heterogeneity and the overall poor methodology of the included studies focusing on BMAC injections. On the other hand, only a few clinical studies analysed the effects of cultured expanded allogenic or autologous BMSCs in humans [81–83]. This is mainly due to the disadvantages of in vitro culture expansion of BMSCs, including lengthy times, risk of contamination, large costs, and legal restriction [84, 85]. This led to the emergence of cell-based products prepared at the point-of-care, capable of bypassing the strict regulations and issues related to cell manipulation and expansion [84].

In this scenario, BMAC has gained a large popularity in clinical practice. Compared with cultured BMSCs, BMAC had the biological advantage to contain also a high number of other cell precursors, platelets rich in growth factors, cytokines, and chemokines which may also play a role in OA prevention [69, 86]. Despite the large use in the clinical practice, preclinical evidence supporting the use of point-of-care bone marrow-derived products is still scarce, as documented in this systematic review. Only 5 studies analysed the disease-modifying effects of point-of-care products in animal OA models, reporting positive effects in 4 studies, while no differences with OA controls were observed in one study [28, 32, 35, 54, 58]. Also, preclinical studies often present important differences in terms of cell source and processing. While most of the preclinical studies presented heterogenous anatomical sources, including femur, tibia, sternum, and humerus, the anterior iliac crest is the most common site used in clinical practice. This could entail important differences, as shown by a study directly comparing BMSCs obtained from iliac crest and proximal tibia in the same patients: Iliac crest BMAC had a four-time higher number of mononucleated cells with significantly higher chondrogenic capacity compared to the tibia [87]. Other differences could be due to the dosage, the injection protocols, as well as the common use of allogeneic cells in animal models, while autologous cells are the preferred choice in humans. Future studies should investigate the most effective preparation method in both preclinical and clinical settings, to optimise the biological potential of the bone marrow source for OA treatment.

This systematic review presents several limitations reflecting those of the available studies. The studies included are characterised by some risk of bias and a high heterogeneity, focusing on different animals and OA models, with different follow-ups, different bone marrow harvest site, and different characteristics of the injected bone marrow-derived products. Due to this high heterogeneity, it was not possible to draw conclusion on the most suitable bone marrow-derived product. Adhering to specific guidelines, for example using the SYRCLE’s tool to reduce the risk of bias when planning a study, would improve the quality and reliability of the results and increase the homogeneity of the studies, thus favouring more in-depth literature analyses. In particular, the models used are an important variable, as all OA animal models are not equal and may entail differences in the analysed outcomes, with their significance in terms of clinical translation still far from being completely elucidated. Moreover, some included studies have only very small group sizes, limiting also the conclusions of those studies. This heterogeneity clearly represents the complexity of this field and explains the difficulties in the literature analysis, study comparisons, and the understanding of some controversial results. Despite these limitations, this systematic review allowed to better quantify the disease-modifying effects of intra-articular injections of bone marrow-derived products for the treatment of OA in preclinical animal studies. These findings are important to understand the potential of bone marrow-derived products and to guide further research to optimise their use in the clinical practice.

## Conclusions

This systematic review of preclinical studies demonstrated that intra-articular injections of bone marrow-derived products can induce disease-modifying effects in the treatment of OA, slowing down the progression of cartilage damage with benefits at macroscopic, histological, and immunohistochemical levels. Positive results have been also observed in terms of clinical and imaging findings, as well as in the change of inflammatory and cartilage biomarkers, while fewer effects have been described on synovial membrane.

## Supplementary Information

Below is the link to the electronic supplementary material.

Supplementary file1 (DOCX 143 KB)
