# Supplementary material for: Cell-based therapies have disease-modifying effects on osteoarthritis in animal models. A systematic review by the ESSKA Orthobiologic Initiative. Part 2: bone marrow-derived cell-based injectable therapies
Source: Knee Surg Sports Traumatol Arthrosc. 2023 Feb 24;31(8):3230–42. doi: 10.1007/s00167-023-07320-3 (PMC10356673; doi:10.1007/s00167-023-07320-3)
Supplement: Supplementary file 1 — Supplementary file1 (DOCX 143 KB) [file 167_2023_7320_MOESM1_ESM.docx]

**Supplementary Table 1**. Characteristics of the included studies on bone marrow derived products.

| **Authors Journal Year** | **Animal  Model** | **Evaluated  Joint** | **OA Model** | **Treatment groups** | **MSCs origin and harvest site** | **Expanded or Point of care** | **MSCs dose** | **Additional procedure** | **Injection Protocol** N. injections Injection timing Injected Volume | **Follow-up** | **Results** |
| --- | --- | --- | --- | --- | --- | --- | --- | --- | --- | --- | --- |
| Bertoni L et al. Int J Mol Sci 2021 [[5](#_ENREF_5)] | 8 Horses | Ferlock joint | Osteochondral chip  fragment creation | BMSCs Umbelical MSCs  OA control | Horse (allogenic) Sternum | Expanded | 1 x 10^7^ cells | N/A | Single injection - 2 ml | 12 weeks | BMSC injections reduced the progression of imaging signs of OA. No differences between umbilical and BMSCs. |
| Huang H et al. Connect Tissue Res 2021 [[36](#_ENREF_36)] | 60 Rabbits | Knee | Fixation  in extension | BMSCs (n=12) BMSCs + Lugua polypeptide (n=12) Lugua polypeptide (n=12) OA control (n=12) Sham contol (n=12) | Rabbit (Allogenic) Unspecified | Expanded | 1 x 10^7^ cells | N/A | Six injections 1-week interval 0.3 ml | 6 weeks | BMSCs combined with Lugua polypeptide injection may improve cartilage tissue damage in OA rabbits. |
| Jin Y et al. J Cell Mol Med 2021 [[39](#_ENREF_39)] | -  Rat | Knee | ACL transection  and medial  meniscectomy | BMSCs Exo-BMSCs OA control Sham control | Unspecified | Expanded | 1 x 10^6^ cells | N/A | Seven injections 1-week interval 100 µL | 8 weeks | Exo and BMSC injections slowed histologic OA progression and improved subchondral bone status. |
| Liu JW et al. Evid Based Complement Alternat Med 2021 [[48](#_ENREF_48)] | 50 Rabbits | Knee | Fixation  in extension | BMSCs (n=10) BMSCs + acupuncture (n=10) Acupuncture (n=10) OA control (n=10) Healthy control (n=10) | Rabbit (Allogenic) Unspecified | Expanded | 1 x 10^6^ cells | N/A | Single injection - 1 ml | 2 weeks | BMSCs treatment slowed OA progression and alleviated pain. The combination with acupuncture enhanced these effects. |
| Xia P et al. Cell Transplantation 2021 [[87](#_ENREF_87)] | 24 Rats | Knee | ACL  transection | BMSCs (n=4) BMSCs + 3-MA (n=4) BMSCs + LIPUS (n=4) BMSCs + 3-MA + LIPUS (n=4) OA control (n=4) Healthy control (n=4) | Rat (Allogenic) Femur | Expanded | 1 x 10^6^ cells | Treatment of MSC  with autophagy  inhibitor | Single injection - 50 µL | 4 weeks | Both BMSCs alone and BMSCs + LIPUS slowed OA progression. LIPUS enhances the cartilage repair effects of BMSCs on OA. |
| Zeng WN et al. Am J Sports Med 2021 [[90](#_ENREF_90)] | 40  Rats | Knee | ACL  transection | BMSCs (n=8) BMSCs + KGN (n=8) KGN (n=8) OA control (n=8) Healthy control (n=8) | Rat (Allogenic) Femur/Tibia | Expanded | 2.5 x 10^6^ cells | Preconditioning with KGN | Two injections 2-week interval 50 µL | 14 weeks | All treatments slowed down OA progression. Combination between BMSCs and KGN increased these effects. |
| Zhang R et al. Cell Transplant 2021 [[91](#_ENREF_91)] | -  Rat | Knee | MIA  injection | BMSCs BMSCs + curcumin OA control | Rat (Allogenic) Femur/Tibia | Expanded | 1 x 10^6^ cells | N/A | Single injection - 50 µL | 4 weeks | Curcumin enhanced the BMSC function.  Both groups alleviated OA progression |
| Zong Z et al. Bone Joint Res 2021 [[92](#_ENREF_92)] | 20 Rabbits | Knee | ACL  transection | BMSCs high dose (n=5) BMSCs medium dose (n=5) BMSCs low dose (n=5) OA Control (n=5) | Human (Xenogeneic) Femur | Expanded | High: 8 x 10^6^ cells Medium: 4 x 10^6^ cells Low: 2 x 10^6^ cells | N/A | Single injection - 100 µL | 8 weeks | Medium and high dose of BMSCs alleviated histological OA progression. |
| Asadi S. et al.  Physiol Int 2020 [[3](#_ENREF_3)] | 63  Rats | Knee | Meniscotibial  ligament  incision | BMSCs (n=7) BMSCs + O_3_ (n=7) BMSCs + exercise (n=7) BMSCs + O_3_ + exercise (n=7) Exercise + O_3_ (n=7) O_3_ (n=7) Exercise (n=7) OA control (n=7) Healthy control (n=7) | Rat (Allogenic) Unspecified | Expanded | 1 x 10^6^ cells/Kg | N/A | Single injection - - | 4 weeks | Combined therapy with exercise, BMSCs and O_3_ significantly increased the expression of SOX9 and HIF1 genes in the cartilage of rats with knee OA. |
| Khatab S et al. Cell Bio Toxicol 2020 [[40](#_ENREF_40)] | 41  Rats | Knee | MIA  injection | BMSCs (n=19) Encapsulated-BMSCs (n=22) Saline (n=19) Tracking study (n=8) | Human (Xenogeneic) Unspecified | Expanded | 1.0 × 10^5^ cells | MSC  encapsulation in alginate beads | Single injection - 25 µL | 8 weeks | BMSCs alleviated pain in the OA model but did not slow OA progression. |
| Olifirenko O et al. Georgian Med News 2020 [[61](#_ENREF_61)] | -  Rabbits | Knee | ACL transection  and medial  meniscectomy | BMAC + PRP PRP OA control Healthy control | Rabbit (autologous) Iliac Crest | Point of care | Unspecified | N/A | Two injections 2-week interval 0.5 ml | 2 months | The adjunct of BMAC to PRP did not improve the results. |
| Shu CC et al. Arthritis Res Ther 2020 [[70](#_ENREF_70)] | 312 Mice | Knee | Meniscectomy | BMSCs (n=78) BMSCs + HA (n=78) HA (n=78) OA control (n=78) | Mouse (Allogenic) Femur/Humerus | Expanded | 2 x 10^4^ cells | N/A | Two injections 2-week interval 10 µL | 12 weeks | BMSCs but not HA significantly reduced late-stage cartilage proteoglycan loss and structural damage. |
| Hamilton AM et al. Plos One 2019 [[33](#_ENREF_33)] | 20 Mice | Knee | Meniscectomy | DiR-Fe-BMSCs (n=5) DiR-BMSCs (n=5) OA control (n=5) Sham control (n=5) | Mouse (Allogenic) Femur/Tibia | Expanded | 5 x 10^4^ cells | Labelling with  fluorescent  DiR or DiR-Fe | Single injection - 4 µL | 4 weeks | BMSCs provided an increase of homeostatic polarized macrophages, reducing pro-inflammatory macrophages in the synovium. |
| Jiang B et al. Theranostics 2019 [[37](#_ENREF_37)] | 8 Monkeys | Knee | Naturaly  occuring | BMSCs (n=3) MSC spheroids (n=3) OA control (n=2) | Monkey (Autologous) Tibia | Expanded | 5 x 10^6^ cells | N/A | Three injections 1-month interval 100 µL | 9 months | Embryonic MSC spheroids and BMSCs improved clinical performance in spontaneous OA in rhesus macaques. |
| Mahmoud EE et al. Bone Joint J 2019 [[51](#_ENREF_51)] | 15 Rabbit | Knee | ACL  transection | Multiple injection BMSCs (n=5) Single injection BMSCs (n=5) OA control (n=5) | Rabbit (Allogenic) Iliac Crest | Expanded | 1 x 10^6^ cells | N/A | Single/Three injections - 400 µL | 2 months | BMSC injections slowed down histological OA progression, with multiple injections having greater effect. |
| McKinney JM et al. Eur Cell Mater 2019 [[56](#_ENREF_56)] | 39 Rats | Knee | Meniscectomy | BMSCs (n=8) Encapsulated-BMSCs (n=8) OA control (n=8) Empty alginite capsules (n=7) Sham control (n=8) | Human (Xenogeneic) Unspecified | Expanded | 5 x 10^5^ cells | MSC  encapsulation | Single injection - 50 µL | 3 weeks | Encapsulated BMSCs showed a chondroprotective role for articular cartilage in early-stage post-traumatic OA |
| Barrachina L. et al. BMC Vet Res 2018 [[4](#_ENREF_4)] | 18 Ponies | Carpal joints | Amphotericin-B  injection | BMSCs-naïve (n = 7) BMSCs-primed (n = 7) OA control (n = 4) | Pony (allogenic) Sternum | Expanded | 2.5 x 10^6^ cells | MSC priming with  TNFα and IFNγ | Two injections 4-month interval 2 ml | 6 months | BMSC treatment alleviated OA progression. BMSC-primed treatment suggested more powerful effects. |
| Cao DN et al. Bio-Des Manuf 2018 [[11](#_ENREF_11)] | 46 Mice | Knee | ACL  transection | BMSCs (n=12) ALK5 BMSCs (n=12) OA control (n=12) | Mouse (Allogenic) Femur/Tibia | Expanded | 1 x 10^7^ cells | Transfection with  ALK5 plasmid | Single injection - 0.1 ml | 12 weeks | BMSCs slowed histological OA progression. ALK transfection improved this effect. |
| Chen YC et al. J Taiwan Inst Chem Eng 2018 [[16](#_ENREF_16)] | 18  Rabbits | Knee | ACL  transection | PRP + BMSCs (n=3) HA + BMSCs (n=3) HA + PRP (n=3) PRP (n=3) HA (n=3) Sham control (n=3) | Rabbit (Autologous) Iliac Crest | Expanded | 5 x 10^6^ cells | N/A | Single injection - 400 μl | 3 months | PRP + BMSCs could be considered as a promising biological method for OA treatment. |
| Desando G et al. Tissue Eng Part C Methods 2018 [[22](#_ENREF_22)] | 24 Rabbits | Knee | ACL  transection | BMSCs (n=12) BMSCs + HA (n=12) BMAC (n=12) BMAC + HA (n=12) HA (n=12) OA control (n=12) Sham control (n=3) | Rabbit (Autologous) Iliac Crest | Expanded or Point of care | 2 x 10^6^ cells  - | N/A | Single injection - 300 µL | 24 weeks | BMSCs and BMC slowed OA progression in the cartilage, synovium, and meniscus. |
| Prasadam I et al. Lab Invest 2018 [[66](#_ENREF_66)] | -  Rats | Knee | Meniscectomy | BMSCs ACCs BMSCs + ACCs HA Sham control | Rat (Allogenic) Femur | Expanded | 3 x 10^5^ cells | N/A | Single injection - 75 µL | 10 weeks | Mixed BMSCs + ACCs treatment was effective in regenerating damaged cartilage in OA disease models. |
| Ude CC et al. Exp Gerontol 2018 [[79](#_ENREF_79)] | 28 Sheep | Knee | ACL transection  and medial  meniscectomy | BMSCs (n=6) ADSCs (n=6) OA control (n=6) | Sheep (Autologous) Iliac Crest | Expanded | 2 x 10^7^ cells | Chondrogenic  lineage induction | Single injection - 5 ml | 48 weeks | Both BMSCs and ADSCs treatments had significantly improved cartilage scores compared to the controls. |
| Wang Z et al. J Orthop Res 2018 [[85](#_ENREF_85)] | 24 Goats | Knee | ACL transection  and medial  meniscectomy | BMAC (n=6) PRP (n=6) Saline (n=6) Control (n=6) | Goat (autologous) Iliac Crest | Point of care | Unspecified | N/A | Three injections 4-week intervals 5 ml | 6 weeks | BMAC inhibited inflammation and prevented progressive cartilage degeneration, with higher effects compared to PRP. |
| Xia TW et al.  Ann Transl Med 2018 [[88](#_ENREF_88)] | 6 Pigs | Knee | Meniscectomy | BMSCs (n=6 joints) OA control (n=6 joints) | Rat (Allogenic) Unspecified | Expanded | 1 x 10^7^ cells | Labelling with  SPIO nanoparticles | Four injections 1-week interval 3 ml | 4 weeks | No therapeutic effect after injection of BMSCs was found. |
| Bhatti FU et al. Osteoarthritis Cartilage 2017 [[6](#_ENREF_6)] | 72 Rats | Knee | ACL transection  and medial  meniscectomy | BMSCs (n=18) BMSCs + Vitamin E (n=18) OA control (n=18) Healthy control (n=18) | Rat (Allogenic) Femur/Tibia | Expanded | 1 x 10^7^ cells | Vitamin E  preconditioning.  Cells labeled with  PKH26 dye. | Single injection - 100 μl | N/A | Vitamin E pretreated BMSCs had an improved ability to impede the progression of OA. |
| Chapman V et al. Stem Cells Int 2017 [[15](#_ENREF_15)] | 79 Rats | Knee | Meniscectomy | Early passage BMSCs (n=10) Late passage BMSCs (n=12) Late passage SiMAG BMSCs (n=11) Kenalog (n=8) OA control (n=22) Sham controls (n=16) | Mouse (Xenogeneic) Unspecified | Expanded | 1.5 x 10^6^ cells | Labelling with  SiMAG | Single injection - 10 μg/ml | 42 days | Neither treatment altered progression of joint pathology nor inflammation. Late passage BMSCs had superior effect on weight-bearing vs early passage BMSCs. |
| Cosenza S et al. SciRep 2017 [[18](#_ENREF_18)] | 75 Mice | Knee | Collagenase  injection | BMSCs (n=15) BMSC Exosomes (n=15) Microparticle (n=15) OA control (n=15) Healthy control (n=15) | Mouse (Allogenic) Unspecified | Expanded | 2.5 x 10^5^ cells | N/A | Single injection - 5 µL | 42 days | BMSCs, Exos, and microparticles exerted similar effect, alleviating OA progression in µCT analysis |
| Peng BY et al. Oncotarget 2017 [[64](#_ENREF_64)] | 12 Mice | Knee | ACL  transection | BMSCs (n=12) OA control (n=6) Sham control (n=6) | Human (Xenogeneic) Unspecified | Expanded | 1x 10^6^ cells | Trasduction wiht  HPV-16 E6/E7 | Single injection - - | 3 months | Histological progression of OA was slowed down by BMSC injections. |
| Chiang ER et al. Plos One 2016 [[17](#_ENREF_17)] | 28 Rabbits | Knee | ACL  transection | BMSCs + HA (n=18 knees) HA (n=18 knees) OA control (n=10 joints) Sham control (n=10 joints) | Rabbit (Allogenic) Femur | Expanded | 1 x 10^6^ cells | Expansion under hypoxic condition | Single injection - 0.4 ml | 12 weeks | Better histological scores were observed with the combination of BMSCs and HA compared to HA alone. |
| Farrell E et al. Stem Cell Res Ther 2016 [[24](#_ENREF_24)] | 40 Mice | Knee | Collagenase  injection | BMSCs (n=8) AdNull BMSCs (n=8) AdIL-10 BMSCs (n=8) Virus AdIL-10 (n=8) OA control (n=8) | Human (Xenogeneic) Iliac Crest | Expanded | 2 x 10^4^ cells | Transduction with  AdIL-10 | Single injection - 6 µL | 6 weeks | No clear reduction in disease onset or progression after BMSCs injection was found. |
| Gupta PK et al. Arthritis Res Ther 2016 [[31](#_ENREF_31)] | 80 Rats | Knee | MIA  injection | High dose BMSCs + HA (n=15) Low dose BMSCs + HA (n=15) HA (n=15) OA control (n=15) Sham (n=6) | Human (Xenogeneic) Iliac Crest | Expanded | High: 1,3 x 10^6^ cells Low: 6 x 10^5^ cells | N/A | Single injection - 30 µL | 12 weeks | BMSCs + HA slowed histological OA progression and alleviated pain. |
| Maumus M et al.  Front Immunol  2016 [[54](#_ENREF_54)] | 60 Mice | Knee | Collagenase  injections | BMSCs (n=10)  Low-dose ASCs (n=10) High-dose ASCs (n=10) Low-dose IFNγ-ASCs (n=10) High-dose IFNγ-ASCs (n=10) OA control (n=10) | Rat (Allogenic) Unspecified | Expanded | 2 x 10^5^ cells | N/A | Single injection - 8 µL | 42 days | Both BMSCs and ASCs revealed protection against cartilage degradation compared to OA controls. |
| Morille M et al. Biomaterials 2016 [[58](#_ENREF_58)] | 24  Mice | Knee | Collagenase  injection | BMSCs-PAM (n=8) BMSCs-PAM-T (n=8) OA Control (n=8) | Human (Xenogeneic) Unspecified | Expanded | 2.5 x 10^5^ cells | Microspheres  releasing TGFb3 | Single injection - 7 µL | 42 days | BMSCs seeded onto PAM-T is a promising tool for endogenous cartilage regeneration. |
| Delling U et al. Cell Transplant 2015 [[21](#_ENREF_21)] | 12 Sheep | Knee | Meniscectomy | SPIO-BMSCs (n=3) BMSCs + SPIO-BMSCs (n=12) Saline (n=4) SPIO (n=2) OA controls (n=3) | Sheep (Autologous) Iliac Crest | Expanded | 2 x 10^7^ cells | Labelling with  SPIO | Single injection - 2 ml | 12 weeks | No significant treatment effects were seen at the conclusion of the study. |
| Lu L et al. J Dent Res 2015 [[50](#_ENREF_50)] | 27 Mice | TMJ | UAC Procedure | BMSCs OA control Healthy control | Unspecified | Expanded | 2 x 10^3^ cells | Labelling with  GFP | Weekly injections for 4, 8, and 12 weeks 20 µL | 12 weeks | Multiple BMSC injections alleviated OA progression in TMJ OA mouse model. |
| Tang JL et al. Mol Med Rep 2015 [[77](#_ENREF_77)] | 36 Rats | Knee | ACL transection  and medial  meniscectomy | BMSCs (n=12) OA control (n=12) Sham control (n=12) | Rat (Allogenic) Femur/Tibia | Expanded | 5 x 10^5^ cells | N/A | Single injection - 0.3 ml | 14 weeks | BMSCs suppressed catabolic factors and slowed down histological OA progression. |
| Ude CC et al. Osteoarthritis Cartilage 2015 [[78](#_ENREF_78)] | 9 Sheep | Knee | ACL transection  and medial  meniscectomy | BMSCs (n=3) ASCs (n=3) OA control (n=3) | Sheep (Autologous) Iliac Crest | Expanded | 4 x 10^6^ cells | Chondrogenic  lineage induction | Single injection - 5 ml | 32 weeks | Both BMSC and ADSC treatments demonstrated the presence of regenerated neo cartilage. |
| Yang X et al. Chin Med J (Engl) 2015 [[89](#_ENREF_89)] | 40 Rats | Knee | ACL transection  and medial  meniscectomy | BMSCs (n=20)  OA control (n=20) | Rat (Allogenic) Femur/Tibia | Expanded | 5 x 10^6^ cells | N/A | Three injections 1-week interval 0.1 ml | 3 weeks | BMSCs slowed OA progression. |
| Caminal M et al. N Biotechnol 2014 [[10](#_ENREF_10)] | 10  Sheep | Knee | Chondral  lesions | BMSCs (n=7) OA control (n=3) | Sheep (Autologous) Sternum | Expanded | 1.2 x 10^7^ cells | N/A | Single injection - 4 ml | 12 months | Better gross and histological grades were observed in animals treated with BMSCs. |
| Jiang L et al. J Tissue Eng Regen Med 2014 [[38](#_ENREF_38)] | 27 Monkeys | Knee | Collagenase  injection | Polyclonal BMSCs (n=9) Clonal BMSCs (n=9) OA control (n=9) | Monkey (Autologous) Humerus | Expanded | 5 x 10^6^ cells | Chondrogenic  lineage induction | Single injection - 150 µL | 24 weeks | Both BMSC treatments improved histological OA scores and gene expression, with best results for selected chondrogenic clonal BMSCs. |
| Kim JE et al. Int J Nanomedicine. 2014 [[41](#_ENREF_41)] | 23 Rats | Knee | ACL transection  and medial  meniscectomy | BMSCs (n=6) SAP - BMSCs (n=6) SAP (n=6) OA control (n=5) | Rat (Allogenic) Femur/Tibia | Expanded | 1 x 10^6^ cells | Encapsulated in  SAP Hydrogel | Single injection - 200 µL | 6 weeks | SAP encapsulated MSCs slowed OA progression in the rat OA model. |
| Singh A et al. Bone Joint Res 2014 [[72](#_ENREF_72)] | 20 Rabbits | Knee | ACL  transection | BMAC (n=10) OA control (n=10) | Rabbit (autologous) Tibia | Point of care | 1 x 10^6^ cells | N/A | Single injection - 1 ml | 20 weeks | BMSCs could be promising cell sources for the treatment of OA. |
| Song Fl et al. Bone Joint Res 2014 [[74](#_ENREF_74)] | 18 Sheep | Knee | ACL transection  and medial  meniscectomy | Concentrated BMSCs (n=6) Expanded BMSCs (n=6) OA control(n=6) Healthy control (n=6) | Sheep (Autologous) Iliac Crest | Expanded | Concentrated:  4.5x10^8^ cells Expanded:  1 x 10^7^ cells | N/A | Single injection - 5 ml | 8 weeks | BMSCs injection slowed OA progression. Expanded cells had better theraputic effect. |
| Ude CC et al. Plos One 2014 [[80](#_ENREF_80)] | 18 Sheep | Knee | ACL transection  and medial  meniscectomy | BMSCs (n=6) ADSCs (n=6) OA control (n=6) | Sheep (Autologous) Iliac Crest | Expanded | 2 x 10^7^ cells | Chondrogenic  lineage induction | Single injection - 5 ml | 6 weeks | Autologous chondrogenically induced BMSCs and ADSCs could be promising cell sources for OA treatment. |
| van Buul GM et al. J Orthop Res 2014 [[81](#_ENREF_81)] | 24 Rat | Knee | MIA injection | Concentrated BMSCs (n=8) Expanded BMSCs (n=8) OA control (n=8) | Rat (Allogenic) Femur/Tibia | Expanded or Point of care | Concentrated:  1x10^6^ cells Expanded:  1 x 10^7^ cells | N/A | Single injection - 50 µL | 4 weeks | No effects of the cellular therapies on structural damage and synovial inflammation were found. |
| Diekman BO et al. Cell Transplant 2013 [[23](#_ENREF_23)] | 30 Mice | Knee | Closed tibial  plateau fracture | BMSCs (n=16) Oa control (n=8) | Mouse (Allogenic) Femur/Tibia | Expanded | 1 x 10^4^ cells | N/A | Single injection - 6 µL | 8 weeks | BMSCs prevented the development of post-traumatic OA. |
| Al Faqeh H et al. Exp Gerontol 2012 [[1](#_ENREF_1)] | 16 Sheep | Knee | ACL transection  and medial  meniscectomy | BMSCs (n=6) Chondrogenic BMSCs (n=6) OA control (n=4) | Sheep (Allogenic) Iliac Crest | Expanded | 2 x 10^6^ cells | Chondrogenic  lineage induction | Single injection - 5 ml | 3 weeks | BMSCs decreased gross OA findings compared to control group. |
| Horie M et al. Osteoarthritis Cartilage 2012 [[35](#_ENREF_35)] | 14 Rats | Knee | Meniscetomy | BMSCs (n=5 joints) BMSCs + cyclopamine (n=5 joints) BMSCs + SAG (n=4 joints) OA control (n=10 joints) | Human and Rat (Xenogeneic) Unspecified | Expanded | 2 x 10^6^ cells | Labelling with  CM-DiI | Single injection - 50 µL | 8 weeks | BMSCs slowed histological OA progression and partially restored the meniscus |
| Sato M et al. Arthritis Res Ther 2012 [[68](#_ENREF_68)] | 60 Guinea pigs | Knee | Naturally  occuring | BMSCs (n=15) BMSCs + HA (n=15) HA (n=15) OA control (n=15) | Human (Xenogeneic) Unspecified | Expanded | 7 x 10^6^ cells | Labelling with  CFDA-SE | Single injection - 2 ml | 5 weeks | BMSCs + HA had better overall results compared with BMSCs alone and OA controls. |
| Suhaeb AM et al. Indian J Exp Biol 2012 [[75](#_ENREF_75)] | 36 Rats | Knee | MIA injection | BMSCs BMSCs + HA HA OA control (n=7) | Rat (Allogenic) Femur | Expanded | 3-5 x 10^6^ cells | N/A | Two injections - 25 µL | 6 weeks | The use of BMSCs and HA can delay the progression of OA. |
| Mokbel AN et al. BMC Musculoskelet Disord 2011 [[57](#_ENREF_57)] | 27 Donkeis | Carpal joint | Amphotericin-B injection | BMSCs + HA (n=27 joints) HA (n=27 joints) | Donkey (autologous) Humerus | Expanded | 1.8-2.3 x 10^6^ cells | Labelling with  GFP | Single injection - 3 ml | 6 months | Better synovial fluid characteristics in BMSCs + HA were found compared to HA alone. |
| Frisbie DD et al. J Orthop Res 2009 [[29](#_ENREF_29)] | 24 Horses | Carpal joint | Osteochondral  defect | BMSCs (n=8) Adipose SVF (n=8) OA control (n=8) | Horse (autologous) Sternum | Expanded | 0.66 x 10^6^ cells | N/A | Single injection - 2 ml | 70 days | BMSCs reduced PGE2 levels in the synovial fluid, but not overall significant benefits were reported. |
| Murphy JM et al. Arthritis and Rheumatism 2003 [[59](#_ENREF_59)] | 24 Goats | Knee | ACL transection  and medial  meniscectomy | BMSCs (n=15) OA control (n=9) | Goat (allogenic) Iliac Crest | Expanded | 2 x 10^6^ cells | Labelling with  GFP | Single injection - 5 ml | 20 weeks | BMSC injection reduced OA progression and stimulated meniscal tissue regeneration. |

3-MA, 3-Methyladenine; ACC, articular cartilage chondrocytes; ACL, anterior cruciate ligament; AdIL-10, adenoviral vectors expressing vIL-10; AdNull, null virus; ALK5, transforming growth factor receptor I; ASCs, adipose-derived stem/stromal cells; BMAC, bone marrow aspirate concentrate; BMSC, bone marrow-derived MSC; CFDA-SE, carboxyfluorescein diacetate succinimidyl ester; CM-Dil, fluorescent dye cell tracker; DiR, fluorescent dye; Exo, exosomes; Fe, ferumoxytol; GFP, green fluorescent protein; KGN, Kartogenin; HA, hyaluronic acid; HIF1, hypoxia-inducible factor-1; HPV, human papilloma virus; IFNγ: interferon gamma; LIPUS, low-intensity pulsed ultrasound; MA, methyladenine; µCT, micro computed tomography; MSCs, mesenchymal stem/stromal cells; O3, ozone; OA, osteoarthritis; PAM-T, microspheres releasing TGFb3; PGE2, prostaglandin E2; PRP, platelet rich plasma; SAG, smoothened agonist; SAP, self-assembled peptide; SOX9, sex-determining region Y box 9; SPIO, superparamagnetic iron oxide particles; SVF, stromal vascular fraction; TNFα, tumor necrosis factor alpha; UAC, unilateral anterior crossbite.
